# Childhood Emotional Abuse and Somatic Symptoms: The Mediating Effect of Self‐Hate

**DOI:** 10.1111/sjop.70008

**Published:** 2025-08-04

**Authors:** Pierre Gilbert Rossini, Francesco Gazzillo, David Kealy

**Affiliations:** ^1^ Department of Clinical and Biological Sciences University of Turin Orbassano Italy; ^2^ Department of Dynamic and Clinical Psychology, and Health Studies Sapienza University of Rome Roma Italy; ^3^ Department of Psychiatry University of British Columbia Vancouver British Columbia Canada

**Keywords:** Control‐Mastery Theory, emotional abuse, self‐hate, somatic symptoms

## Abstract

Childhood emotional abuse is a recognized factor for long‐term psychological and physical health problems, including persistent somatic symptoms. Negative self‐beliefs, such as self‐hate‐related beliefs, may explain how emotional mistreatment contributes to physical distress in adulthood beyond general emotional difficulties. A longitudinal study was conducted with 298 help‐seeking adults. At baseline, participants completed measures of childhood emotional abuse, self‐hate, and general negative affectivity. Somatic symptoms were assessed two months later. Mediation analyses tested whether self‐hate mediated the relationship between childhood emotional abuse and somatic symptoms while accounting for the influence of general negative affectivity, which reflects a general tendency to experience negative emotional states. Participants (*M*_age_ = 33.7, SD = 11.8) were predominantly female (63.3%) and Caucasian (84.2%). Childhood emotional abuse (*M* = 11.24, SD = 6.60) was significantly associated with self‐hate (*M* = 10.35, SD = 6.13, *r* = 0.33, *p* < 0.001) and somatic symptoms (*M* = 13.99, SD = 6.76, *r* = 0.27, *p* < 0.001). Self‐hate strongly correlated with somatic symptoms (*r* = 0.45, *p* < 0.001). Mediation analyses showed that self‐hate significantly mediated the link between emotional abuse and somatic symptoms, even after accounting for general negative affectivity as a covariate (indirect effect = 0.07, 99% CI [0.03, 0.13]). These findings highlight self‐hate as a key mechanism linking childhood emotional abuse to somatic symptoms. Even when controlling for broader emotional instability, self‐hate remained central. Addressing these negative self‐beliefs through targeted interventions may help reduce both emotional suffering and associated physical manifestations.

**Summary**

- Childhood emotional abuse predicts somatic symptoms in adulthood among help‐seeking individuals.
- Self‐hate mediates the link between childhood emotional abuse and somatic complaints, even when controlling for general negative affectivity.
- Self‐hate shows a stronger correlation with somatic symptoms than emotional abuse alone and emerges as an independent, clinically relevant construct.
- Targeting self‐hate in therapy may reduce both psychological suffering and physical symptoms.

## 1 Introduction

Childhood mistreatment is a well‐documented risk factor for the development of both psychological and physiological health issues (Felitti et al. 1998; Nemeroff 2016). Among different forms of mistreatment, emotional abuse is often underestimated. It involves a pattern of behavior by caregivers or authority figures that undermines a child's emotional well‐being and self‐esteem through criticism, humiliation, unrealistic expectations, unfair treatment, or unresponsiveness to developmental needs (Kumari 2020). Research indicates that individuals who experience mistreatment in early life are more likely to report significant physical symptom distress as adults, including pain, gastrointestinal issues, and other psychosomatic complaints (Eilers et al. 2023). These findings highlight the lasting impact of early adversities on both psychological and physiological health, particularly in relation to somatic symptoms in later life (Cay et al. 2022).

Functional somatic disorders (Löwe et al. 2024), including conversion and somatic symptom disorders (SSD), are particularly relevant for understanding the long‐term consequences of childhood adversity. These disorders involve persistent and distressing physical symptoms without a clear medical explanation, often occurring alongside significant psychological distress. This association reflects the interplay between early‐life trauma and later physical health, suggesting that unresolved emotional pain from adverse experiences may manifest in physical symptoms (Löwe et al. 2024). Consistent with this, individuals with SSD frequently report histories of childhood adversity, indicating the association between emotional mistreatment in early life and somatic symptom distress in adulthood (Kuhar and Zager Kocjan 2022).

One pathway through which childhood mistreatment may lead to somatic symptom distress is the formation of negative self‐beliefs and maladaptive emotional patterns. Previous studies have highlighted the roles of shame and guilt in this process, suggesting that negative attributions toward oneself, inherited from traumatic experiences, may mediate the association between trauma and somatic symptoms (Kealy et al. 2018). Indeed, mistreated children often internalize harsh and rejecting attitudes from caregivers, developing a persistent sense of worthlessness and generalized “bad me” shame (Benau 2017), which may manifest—potentially later in life—through physical correlates. These symptoms can represent unprocessed emotional pain, arising when shame becomes overwhelming and cannot be symbolized or mentalized (Lumley et al. 2011). However, much of this research relies on cross‐sectional data, leaving unclear causal and dynamic relationships between these variables.

Additionally, no study has specifically examined self‐hate, a self‐representation defined by persistent beliefs of being inherently worthless, broken, and undeserving of love, appreciation, or protection (Leonardi et al. 2022). While related to broader constructs like negative affectivity—which reflects a general tendency to experience negative emotions such as anxiety or sadness (Widiger and Oltmanns 2017)—self‐hate is distinct in both its stability and specificity. It represents a uniquely hostile and self‐directed attitude, often rooted in particularly adverse and repetitive early‐life experiences, such as emotional abuse or neglect. These experiences foster the internalization of critical or rejecting attitudes from caregivers or authority figures, ultimately reinforcing a flawed and unworthy self‐concept (Gilbert et al. 2004; Leonardi et al. 2022).

The present study was aimed at further examining the link between childhood mistreatment and somatic symptoms, with particular attention to the role of self‐hate. Specifically, we hypothesized that self‐hate would mediate the emotional abuse‐somatic symptom relationship, and that the effect of self‐hate would account for this association beyond the effect of general negative affectivity. To overcome concerns about common method variance in previous cross‐sectional studies, somatic symptoms were assessed at a second time point, separated by two months, from emotional abuse and self‐hate. General negative affectivity was controlled for across the mediation model. Furthermore, in the interest of translating findings to clinical populations, we investigated our hypothesized model in a sample of individuals with recent mental health concerns.

## 2 Materials and Methods

### 2.1 Participants and Procedures

A sample of help‐seeking participants was sourced online through the Prolific (https://www.prolific.com/) recruitment platform. Inclusion criteria were being at least 18 years old, based in the UK, and having recently sought mental health care through the National Health Service (NHS). Participants received modest payments of £7.00. A total of 356 individuals consented to participate. Data screening removed 5 incomplete and 19 inattentive responders (e.g., failure of at least two out of three attention check items). A further 34 participants did not complete the second wave of assessment, resulting in a final sample of 298 for the present study. Study questionnaires were completed online using the Qualtrics program. Questionnaires assessing perceived childhood emotional abuse, self‐hate, and general negative affectivity were completed at time 1, and the questionnaire assessing somatic symptoms was completed two months later at time 2. Ethics approval for the study was provided by the last author's university Behavioral Research Ethics Board; all participants provided informed consent to take part in the study.

Participants' average age was 33.73 years (SD = 11.81; range = 18–74). Nearly two‐thirds, 63.3%, identified as female; 32.6% as male; and 4.3% as non‐binary gender identity. The majority, 84.2%, identified as Caucasian; 3.4% identified as Asian; 3% as South Asian; with the remaining subjects indicating other or mixed ethnicities. Regarding sexual orientation, 64.8% identified as heterosexual, with 21.5% identifying as bisexual, 4.7% as gay or lesbian, and 8.9% as queer or other identities. Most participants, 85.6%, were educated beyond high school, and more than half, 60.1%, were employed; 15.8% were students.

### 2.2 Measures

Perceived childhood emotional abuse was assessed using the 7‐item Emotional Abuse subscale of the Child Abuse and Trauma Scale (CATS‐EA; Kent and Waller 1998; Sanders and Becker‐Lausen 1995). The CATS‐EA uses items such as “Did your parents insult you or call you names?” scored from 0 (never) to 4 (always), with higher scores indicating recollections of more frequent childhood emotional abuse. Strongly correlated with other measures of emotional abuse and aggression (Baker and Festinger 2011), the CATS‐EA demonstrated excellent internal consistency in the present sample (*α* = 0.93).

Self‐hate was assessed using the 5‐item Hated Self subscale of the Forms of Self‐Criticizing/Attacking & Self‐Reassuring Scale (FSCRS; Gilbert et al. 2004). The FSCRS refers to individuals' typical responses toward the self when encountering setbacks or mistakes. Responses to items (e.g., “I do not like being me”) are scaled from 0 (*not at all like me*) to 4 (*extremely like me*), with higher scores indicating more severe hate directed toward the self. Strong internal consistency (*α* = 0.91) was demonstrated in the present sample.

General negative affectivity was assessed using the neuroticism subscale from a brief instrument of the International Personality Item Pool (mini‐IPIP—Donnellan et al. 2006). This 4‐item subscale (e.g., “In general I have frequent mood swings”) is scored from 1 (*strongly disagree*) to 5 (*strongly agree*), with higher scores reflecting a greater tendency to experience negative or unstable affect. This brief neuroticism measure showed acceptable internal consistency (*α* = 0.71) in the present sample.

The 8‐item Somatic Symptom Scale–8 (SSS‐8; Gierk et al. 2014) was used to measure a range of somatic symptoms, including cardiovascular symptoms, gastrointestinal symptoms, pain, and general malaise experienced over the previous 7 days. The SSS‐8 uses a 5‐point scale from 0 (*not at all*) to 4 (*very much*), where higher scores indicate more severe somatic symptom burden. This scale performs comparably with other somatic symptom measures (Toussaint et al. 2016), with high scores predicting increased health care visits (Gierk et al. 2014). The SSS‐8 demonstrated good internal consistency in the present sample (*α* = 0.83).

### 2.3 Analytic Approach

Analyses were performed using SPSS 29 and PROCESS 4.2 (Hayes 2022). Zero‐order correlations were computed to examine associations among study variables. Next, linear regression analyses were conducted to examine the hypothesized mediation model. The model included childhood emotional abuse as the independent variable, somatic symptoms as the dependent variable, and self‐hate as the mediator variable. General negative affectivity was entered as a control variable in each path. The indirect effect of childhood emotional abuse was evaluated conservatively using bootstrapped 99% confidence intervals (CIs), sampled 10,000 times, with an absence of zero within the CI indicating significant mediation (*p* < 0.01). Follow‐up analyses tested age and gender as covariates.

## 3 Results

**FIGURE 1:**
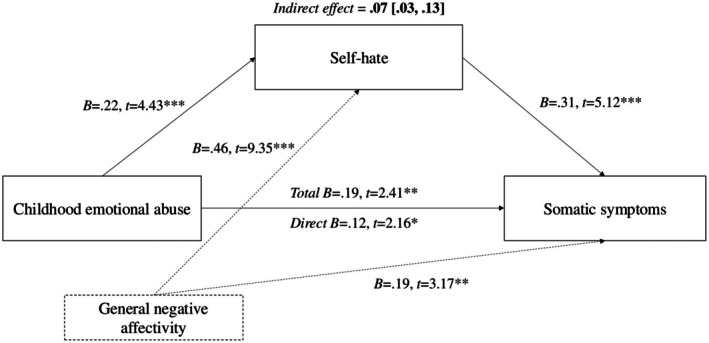
Standardized coefficients for regression analyses testing self‐hate as a mediator of perceived childhood emotional abuse and somatic symptoms. *p < 0.05; **p < 0.01; ***p < 0.001.

Demographic and clinical characteristics of the sample are displayed in Table 1. Standardized coefficients for the regression analyses are included in the Supporting Information. Zero‐order correlations, presented with descriptive statistics in Table 2, indicated significant positive associations between all study variables. Regression analyses indicated a significant total effect of childhood emotional abuse on somatic symptoms, controlling for general negative affectivity, *F* (2, 295) = 31.34, *p* < 0.001, *R* ^2^ = 0.18, as well as significant paths from childhood emotional abuse to self‐hate, *F* (2, 295) = 67.08, *p* < 0.001, *R* ^2^ = 0.31, and from self‐hate to somatic symptoms, *F* (3, 294) = 31.42, *p* < 0.001, *R* ^2^ = 0.24, with the inclusion of emotional abuse and negative affectivity. Standardized coefficients and t‐tests for variables in the model are presented in Figure 1. Analysis of the indirect effect of childhood emotional abuse indicated a standardized point estimate of 0.07, 99% CI [0.03, 0.13], with an absence of zero within these bounds. Thus, consistent with our hypothesis, self‐hate was a significant mediator of the relation between childhood emotional abuse and somatic symptoms, controlling for general negative affectivity (which was significant in each path). Follow‐up analyses, testing age and gender as covariates, revealed no change to the significance or size of the mediation effect, and hence for the sake of parsimony, these variables were not retained in the final model.

**TABLE 1:** Socio‐demographic characteristics and self‐reported somatic symptoms (*N* = 298).

Variable		
Age	*M*	SD
Years	33.73	11.81
Gender	*n*	%
Female	188	63.1
Male	97	32.6
Non‐binary or non‐conforming	13	4.3
Ethnic identity	*n*	%
Caucasian	251	84.2
Asian	10	3.4
South Asian	9	3
African	6	2
Multiple or other ethnicities	22	7.4
Sexual orientation	*n*	%
Heterosexual	193	64.8
Gay or lesbian	14	4.7
Bisexual	64	21.5
Asexual	7	2.3
Queer or other orientation	20	6.6
Highest level of education	*n*	%
High school or equivalent	42	14.1
Some college, no degree	77	25.8
Technical or trade diploma	24	8.1
University degree	155	52
Employment	*n*	%
Full‐time employment	124	41.6
Part‐time employment	55	18.5
Student	47	15.8
On disability benefits	32	10.7
Stay‐home parent	11	3.7
Retired	5	1.7
Not employed	24	8
Somatic symptom frequency, past 7 days (Time 2); range 0–4	*M*	SD
Stomach or bowel problems	1.68	1.28
Back pain	1.74	1.34
Pain in arms, legs, or joints	1.41	1.29
Headaches	1.70	1.31
Chest pain or shortness of breath	1.10	1.20
Dizziness	1.01	1.09
Feeling tired or low energy	2.88	1.18
Trouble sleeping	2.48	1.35

**TABLE 2:** Descriptive statistics and zero‐order correlations regarding perceived childhood emotional abuse, self‐hate, general negative affectivity, and somatic symptoms.

	*M* (SD)	1	2	3
1. Childhood emotional abuse (time 1)	11.24 (6.60)	—		
2. Self‐hate (time 1)	10.35 (6.13)	0.33*	—	
3. General negative affectivity (time 1)	3.76 (0.82)	0.24*	0.52*	—
4. Somatic symptoms (time 2)	13.99 (6.76)	0.27*	0.45*	0.38*

* *p* < 0.001.

## 4 Discussion

The results of this study provide empirical support for the hypothesis that self‐hate plays a central mediating role in the relationship between childhood emotional abuse and adult somatic symptom distress.

Consistent with prior literature, we found that childhood emotional abuse was significantly associated with higher levels of self‐hate. This reinforces the notion that emotionally invalidating environments—characterized by chronic criticism, neglect, or rejection—profoundly disrupt the development of a coherent and positive sense of self (Gilbert et al. 2004; Leonardi et al. 2022). These environments can foster enduring negative core beliefs—such as “I am unworthy” or “I am defective”—that may persist into adulthood and provide a cognitive‐emotional foundation for chronic self‐directed hostility (Zhang et al. 2023). In line with this, early adverse experiences have been linked to both psychological and physical health burdens later in life (Kuhar and Zager Kocjan 2022).

Crucially, both childhood emotional abuse and self‐hate independently predicted the severity of somatic symptoms. This finding is in line with biopsychosocial models that link early emotional trauma to long‐term dysregulation in neuroendocrine and immune systems (Lumley et al. 2011; Slavich 2020). However, the independent effect of self‐hate suggests that it is not merely a byproduct of trauma but a key mechanism through which early adversity can be embodied. Through processes of emotional suppression, internal criticism, and self‐punishment, self‐hate may act as a persistent internal stressor that exacerbates both psychological and physical symptoms (Gilbert and Basran 2019).

Further reinforcing this interpretation, our analyses showed that self‐hate was more strongly associated with somatic symptoms than either emotional abuse or general negative affectivity. This distinction is important: while negative affectivity reflects a broad emotional vulnerability (Widiger and Oltmanns 2017), self‐hate captures a more specific and enduring form of self‐directed aggression. This may help explain why self‐hate, rather than general mood instability, emerges as a more robust predictor of somatic symptomatology. These findings resonate with trauma‐informed and psychodynamic theories, which posit that unprocessed emotional pain can be expressed through bodily symptoms in the absence of conscious awareness or verbal elaboration (Gabbard 2000; van der Kolk 2014). Although not the focus of the present study, this finding is also compatible with research on dissociative processes, which emphasizes how early adversity can fragment the integration of cognitive, emotional, and bodily experiences (Schauer and Elbert 2010; McHugh and Egan 2023). In this context, it is possible that self‐hate may be linked to dissociative mechanisms, where unresolved emotional pain could be displaced onto the body, where it may manifest in a chronic, embodied form that is less accessible to verbal processing (Lumley et al. 2011).

Mediation analyses further confirmed that self‐hate significantly mediates the relationship between emotional abuse and somatic symptoms, even after controlling for general emotional distress. This finding aligns with the CMT framework (Weiss 1993; Silberschatz 2005), which conceptualizes maladaptive self‐hate beliefs as defensive adaptations to early unsafe environments. When these beliefs are maintained unconsciously, they can organize emotional and somatic functioning in adulthood, often in ways that reinforce suffering and inhibit healing (Gazzillo 2023). CMT also emphasizes that somatic symptoms can serve as symbolic expressions of self‐punishment, reflecting beliefs of inadequacy and unworthiness (Gazzillo et al. 2024).

From a clinical perspective, these findings emphasize the need to explicitly assess and address internalized self‐hate in individuals with histories of emotional abuse who present with somatic symptoms. By identifying and challenging maladaptive beliefs, therapy enables patients to experience corrective emotional experiences, process repressed emotions, and develop more compassionate and adaptive self‐perceptions (Silberschatz 2005; Gilbert 2020). Thus, addressing self‐hate could potentially alleviate not only psychological distress but also the physical symptom burden associated with unresolved trauma.

This study is not exempt from some limitations. The sample, consisting of online help‐seeking individuals, may limit the applicability of the findings to broader or non‐clinical populations. Additionally, cultural and socioeconomic factors that could influence experiences of abuse and self‐perception were not explicitly addressed. The reliance on self‐reported data introduces potential biases, such as recall errors or social desirability effects, which may affect the results. While the longitudinal design strengthens the study, the relationships observed remain correlational. Future research should consider diverse populations, including younger individuals and non‐clinical groups, and adopt methodologies such as ecological momentary assessment or experimental designs to explore causal relationships.

## 5 Conclusion

These findings support the theoretical contention that self‐hate—distinct from general negative affectivity—can account for the link between childhood emotional abuse and somatic symptoms experienced in adulthood. This knowledge can support clinicians' assessment and case formulation when working with individuals who present with prominent psychosomatic symptoms, and who report maltreatment in their developmental history. Indeed, the findings point to the disconfirming of a negative and broken self‐image—potentially via a corrective therapeutic relationship or specific technical strategies—as a key aspect in reducing the lasting impact of emotional abuse and its somatic manifestations.

## Author Contributions

**Pierre Gilbert Rossini:** conceptualization, writing – original draft, writing – review and editing. **Francesco Gazzillo:** conceptualization, writing – review and editing. **David Kealy:** conceptualization, writing – original draft, data management and collection, writing – review and editing.

## Ethics Statement

The procedures conducted in this study have received approval from the Behavioral Research Ethics Board of the University of British Columbia, with protocol number H20‐02776. Furthermore, the study adhered to the principles set forth in the 1964 Helsinki Declaration and its subsequent amendments, as well as equivalent ethical standards.

## Consent

All participants provided their informed consent through the Qualtrics platform.

## Conflicts of Interest

The authors declare no conflicts of interest.

## Supporting information


Data S1.

## Data Availability

The data that support the findings of this study are available on request from the corresponding author. The data are not publicly available due to privacy or ethical restrictions.
