# Supplementary material for: Childhood Emotional Abuse and Somatic Symptoms: The Mediating Effect of Self‐Hate
Source: Scand J Psychol. 2025 Aug 4;66(6):1005–10. doi: 10.1111/sjop.70008 (PMC12611229; doi:10.1111/sjop.70008)
Supplement: Supplementary file 1 — Data S1. [file SJOP-66-1005-s001.docx]

**Supplementary Material**

Standardized coefficients for regression analyses testing self-hate as a mediator of perceived childhood emotional abuse and somatic symptoms, including socio-demographic covariates*.*

|  | β | *t* | *p* |
| --- | --- | --- | --- |
| *Predicting Self-hate* |  |  |  |
| Emotional abuse | .21 | 4.13 | **<.001** |
| Neuroticism | .47 | 9.60 | **<.001** |
| Age | -.16 | -3.37 | **<.001** |
| Gender | -.06 | -1.21 | .226 |
| *Predicting Somatic Symptoms, Time 2* |  |  |  |
| Emotional abuse | .10 | 1.84 | .066 |
| Self-hate | .32 | 5.15 | **<.001** |
| Neuroticism | .18 | 2.99 | **.003** |
| Age | .01 | .09 | .926 |
| Gender | .12 | 2.23 | **.027** |
|  | Effect | 99% CI | |
| Indirect effect of Emotional abuse on Somatic Symptoms |  |  |  |
| *through Self-hate* | .07 | **.020** | **.124** |

*Note:* Boldface indicates statistical significance. Gender is coded 1=female.
